# Analysis of a Multiple Delays Model for Treatment of Cancer with Oncolytic Virotherapy

**DOI:** 10.1155/2019/1732815

**Published:** 2019-09-30

**Authors:** Adil El Alami laaroussi, Mohamed El Hia, Mostafa Rachik, Rachid Ghazzali

**Affiliations:** ^1^Laboratory of Analysis Modeling and Simulation (LAMS), Department of Mathematics and Computer Science, Faculty of Sciences Ben M'Sik, Hassan II University, Mohammedia, BP 7955, Sidi Othman, Casablanca, Morocco; ^2^Laboratory of Modeling Applied to Economy and Management (LMAEGE), Faculty of Law, Economics and Social Sciences Ain Sebaa Casablanca, Hassan II University, Casablanca, Morocco

## Abstract

Despite advanced discoveries in cancerology, conventional treatments by surgery, chemotherapy, or radiotherapy remain ineffective in some situations. Oncolytic virotherapy, i.e., the involvement of replicative viruses targeting specific tumor cells, opens new perspectives for better management of this disease. Certain viruses naturally have a preferential tropism for the tumor cells; others are genetically modifiable to present such properties, as the lytic cycle virus, which is a process that represents a vital role in oncolytic virotherapy. In the present paper, we present a mathematical model for the dynamics of oncolytic virotherapy that incorporates multiple time delays representing the multiple time periods of a lytic cycle. We compute the basic reproductive ratio *R*_0_, and we show that there exist a disease-free equilibrium point (DFE) and an endemic equilibrium point (DEE). By formulating suitable Lyapunov function, we prove that the disease-free equilibrium (DFE) is globally asymptotically stable if *R*_0_ < 1 and unstable otherwise. We also demonstrate that under additional conditions, the endemic equilibrium is stable. Also, a Hopf bifurcation analysis of our dynamic system is used to understand how solutions and their stability change as system parameters change in the case of a positive delay. To illustrate the effectiveness of our theoretical results, we give numerical simulations for several scenarios.

## 1. Introduction

The continuous improvement of conventional treatments in cancerology (surgery, chemotherapy, and radiotherapy) allows for a major progress in the fight against cancer. Nevertheless, in some situations, these modes of therapy may be ineffective. The development of new therapeutic strategies, therefore, appears essential in order to improve the healing of this disease. Thus, in the last decades, virotherapy of cancers appears to be a credible alternative to some situations, due to advanced discoveries and accurate informations about viruses and also the production of recombinant viral vectors which can be used in cancer gene therapy. The use of replicative viruses as antitumor therapeutic agents (oncolytic viruses) is based on the idea that they reproduce preferentially within the tumor cells; however, normal cells remain immune to infection. These viruses (oncolytic viruses) are either virus with a natural ability to replicate preferentially within tumor cells or viruses genetically modified to hold this property. Genetic modifications are primarily used to improve the specificity of viruses against tumor cells, by targeting a particular surface molecule, by deleting specific viral genes required for replication in healthy cells, or by using activatable viral promoters only in tumor cells [1]. Using viruses to treat cancer is not a new concept. Viruses have attracted interest as anticancer therapeutics since the beginning of the 20th century. However, for several years, research in this field was limited due to technological limitations. In the last 30 years, by increasing understanding of the nature of viruses, their mechanisms of oncolytic activity and their ability to manipulate and exploit genetically has prompted a new wave of oncolytic virotherapy. Today, there is extensive literature describing progress in both theoretical and clinical trials of oncolytic viruses. For more details, we refer the interested reader to [2–7].

Mathematical modeling of oncolytic virotherapy can illuminate the underlying dynamics of treatment systems and lead to optimal treatment strategies. Several studies have been the subject of the study of virotherapy. The first mathematical models of oncolytic viral therapy used ordinary differential equations to describe the fundamental interactions between two types of tumor cells (infected cells and uninfected cells) [8, 9]. Other works consider spatial representation of tumors [10, 11, 12], multiscale effects [13], and stochastic processes [14]. For a review of different mathematical modeling approaches ranging from ordinary differential equations to spatially explicit agent-based models, see [15–17].

Biological experiments helped to understand and explain the lytic cycle, which takes place in six stages; the six stages are as follows: attachment, penetration, transcription, biosynthesis, maturation, and lysis. To infect a new cell, a virus must penetrate inside the cell through the plasma membrane; the virus attacks a receptor on the cell membrane and then releases its genetic material in the cell. In the third step, the host cell's DNA is degraded and the cell's metabolism is directed to initiate the fourth step; biosynthesis, here the virus uses cellular mechanisms to constitute a large amount of viral components and, in the meantime, destroys the DNA of the host cell. Then, it enters the last two stages, maturation and lysis. When many copies of viral are manufactured, they are assembled into complete formed viruses. About 25 minutes after initial infection, approximately 200 new bacteriophages (virions) are formed. Once enough virions have matured and accumulated, specialized viral proteins are used to dissolve the bacterial cell wall, where they can go on to infect other cells and another lytic cycle begins (for more details on the lytic cycle, see [5, 18]). In this work, the dynamics of oncolytic virotherapy are studied by incorporating the viral lytic cycle time. The duration of the intracellular viral life cycle is an essential factor in viral therapy. For example, some viruses require only 30 minutes, some viruses take several hours to complete this process, and some may take days [19]. Therefore, it is necessary and realistic to consider and taking into account the cycle time in modeling the oncolytic virotherapy which allows us to better predict its dynamics. We construct a mathematical model of virotherapy with multiple delays representing the six time periods of the lytic cycle; it is assumed that the time of each stage of the lytic cycle is constant.

Several studies have studied and analyzed systems of delayed differential equations that model virotherapy. In the paper entitled “Hopf Bifurcation Analysis in a Delayed System for Cancer Virotherapy” [20], the authors consider a delayed differential equation system. In [19], Wang et al. propose a mathematical model for oncolytic virotherapy where they consider the time period of the viral lytic cycle as a delay parameter. The novelty of our work is modeling the variation of duration in the intracellular viral life cycle by adding multiple delays; each one represents the time period of each stage of the lytic cycle. We compute the basic reproductive ratio *R*_0_, and we show that there exist a disease-free equilibrium point (DFE) and an endemic equilibrium point (DEE). By formulating suitable Lyapunov function, we prove that the DFE is globally asymptotically stable if *R*_0_ < 1 and unstable otherwise. We also demonstrate that under additional conditions, the DEE is stable. Furthermore, a bifurcation analysis of our dynamical system is used to understand how solutions and their stability change as the parameters in the system vary. To illustrate our theoretical results, numerical simulations are also presented for several scenarios.

This paper is organized as follows. In Section 2, we present our mathematical model. In Section 3, we compute the equilibrium of our model and investigate its stability. Following that, in Section 4, a bifurcation analysis of the dynamical system is used to understand how the solutions and their stability change as the parameters change. Numerical studies are shown, in Section 5, to validate the analytical results. Finally, we conclude the paper in Section 6.

## 2. The Basic Mathematical Model

In a previous work [21], we analyzed the stability of a nonlinear system of differential equations based on the models proposed by [22, 23]. Our model contains three variables, which are, uninfected tumor cell population *x*(*t*), infected tumor cell population *y*(*t*), and free virus particles which are outside cell *v*(*t*), and it has the following form:

(1) $$\begin{array}{c} \left\{\begin{array}{c} \frac{dx\left(t\right)}{dt}=rx\left(t\right)\left(1-\frac{x\left(t\right)+y\left(t\right)}{K}\right)-\beta x\left(t\right)v\left(t\right)-\rho x\left(t\right)y\left(t\right),\\ \frac{dy\left(t\right)}{dt}=\beta x\left(t\right)v\left(t\right)-\delta y\left(t\right),\\ \frac{dv\left(t\right)}{dt}=b\delta y\left(t\right)-\gamma v\left(t\right)-\beta x\left(t\right)v\left(t\right), \end{array}\right. \end{array}$$

where *x*(0)=*x*_0_, *y*(0)=*y*_0_, and *v*(0)=*v*_0_ are given.

The term *rx*(1 − ((*x*+*y*)/*K*)) describes the logistic growth rate of an uninfected tumor cell population *x*(*t*). The constant *r* > 0 is the growth rate, with *K* being the carrying capacity or maximal tumor size so that *x*+*y* ≤ *K*. The term *βxv* represents the rate of infected cells by free virus *v*(*t*), with *β* > 0 being the corresponding constant rate. The term *ρxy* models infection from an encounter between an infected cell and an uninfected cell resulting in cell fusion that produces a syncytium, with *ρ* > 0 being the constant rate describing cell to cell fusion with the formation of syncytia. Infected cells die at a rate of *δy*, and *γv* is the rate of elimination of free virus particles by various causes including nonspecific binding and generation of defective interfering particles. Its burst size models the virus replication ability, the burst size of a virus, which is an essential parameter of virus reproduction. So, our model includes also a parameter *b* that models the burst size.

**Figure 1:**
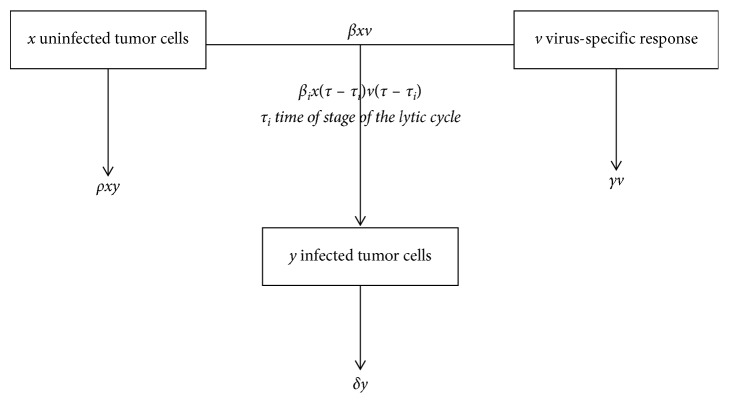
Schematic diagram of the model for virotherapy.

As we mentioned in Introduction, the model (1) did not take into account the time needed to complete the lytic cycle. As a reminder, the lytic cycle is the process where a virus overtakes a cell and uses the cellular machinery of its host to reproduce. Copies of the virus fill the cell to bursting, killing the cell and releasing viruses to infect more cells. The duration of this process varies from virus and more cells. Wang et al. [19] proposed a model of virotherapy with a single delay time; the originality of our work is to make a generalization by introducing 6 delays representing each period of stages of the lytic cycle in order to describe a more realistic situation because the virus goes through 6 stages of life and each one of them may have a different delay. We denote *τ*_*i*_(*i*=1,2,3,4,5,  *n*=6), the different times period of the lytic cycle. The rate of change of infected tumor cells at time *t* will be determined by the tumor cell population and free virus at time *t* − *τ*_*i*_, namely, *x*(*t* − *τ*_*i*_)*v*(*t* − *τ*_*i*_); for more details about the lytic cycle, see Figure 1. Therefore, the model we propose is given as follows:

(2) $$\begin{array}{c} \left\{\begin{array}{c} \frac{dx\left(t\right)}{dt}=rx\left(t\right)\left(1-\frac{x\left(t\right)+y\left(t\right)}{K}\right)-\beta x\left(t\right)v\left(t\right)-\rho x\left(t\right)y\left(t\right),\\ \frac{dy\left(t\right)}{dt}=\overset{n}{\underset{i=1}{\sum }}\beta_{i}x\left(t–\tau_{i}\right)v\left(t–\tau_{i}\right)-\delta y\left(t\right),\\ \frac{dv\left(t\right)}{dt}=b\delta y\left(t\right)-\gamma v\left(t\right)-\beta x\left(t\right)v\left(t\right), \end{array}\right. \end{array}$$

where *τ*_0_=0, *τ*_1_ < *τ*_2_ < ⋯<*τ*_*n*_, and *β*=∑_*i*=1_^*n*^*β*_*i*_.

Using the Van Den Driesseche and Watmough next-generation approach, we calculate the basic reproductive ratio of system (2), which leads to

(3) $$\begin{array}{c} R_{0}=\frac{\beta Kb}{\beta K+\gamma }. \end{array}$$

This parameter plays a major role in our analysis. It represents the number of new virus particles generated by a single virus particle that is inserted into a tumor consisting entirely of uninfected tumor cells [12].

## 3. Model Analysis

In this section, we show the existence of the equilibrium points and we study their stabilities. System (2) has three equilibrium, *E*_0_=(0,0,0), *E*_1_=(*K*, 0,0), and the positive equilibrium *E*^*∗*^(*x*^*∗*^, *y*^*∗*^, *v*^*∗*^), where

(4) $$\begin{array}{c} x^{*}=\frac{\gamma }{\beta \left(b-1\right)},\\ y^{*}=\frac{\gamma r\left(Kb\beta -K\beta -\gamma \right)}{\beta \left(b-1\right)\left(r\gamma +\delta K\beta \left(b-1\right)+\rho K\gamma \right)},\\ v^{*}=\frac{\delta r\left(Kb\beta -K\beta -\gamma \right)}{\beta \left(r\gamma +\delta K\beta \left(b-1\right)+\rho K\gamma \right)}. \end{array}$$

The Jacobian matrix of system (2) at an arbitrary point is given by

(5) $$\begin{array}{c} J=\left(\begin{array}{ccc} -\frac{rx^{*}}{K} & -\left(\rho +\frac{r}{K}\right)x^{*} & \beta x^{*}\\ \overset{n}{\underset{i=1}{\sum }}\beta_{i}v^{*}e^{\left(-\lambda \tau_{i}\right)} & -\delta & \overset{n}{\underset{i=1}{\sum }}\beta_{i}x^{*}e^{\left(-\lambda \tau_{i}\right)}\\ -\beta v^{*} & b\delta & -\gamma -\beta x^{*} \end{array}\right). \end{array}$$

The first equilibrium + represents the total success of therapy. It is easy to prove that *E*_0_ is always unstable. Biologically, the instability of this equilibrium is because, in the absence of the virus, the number of infected cells *y* will remain at 0, and tumor cell population increases. The second equilibrium *E*_1_ represents the failure of virotherapy, as the tumor achieved its maximal size *K*. The partial success of virotherapy is represented by the third equilibrium *E*^*∗*^. The approach that we use to prove the stability of the steady states is divided into two parts: the first one concerns the necessary condition of stability when there is no delay *τ*_*i*_=0 for (*i*=1,…, *n*). In the second step, we prove that the matrix (5) does not have any imaginary eigenvalue. However, in our case, it was not easy to apply the classical theorems of stability because we deal with a system with multiple discrete delays as a summation ∑_*i*=1_^*n*^*β*_*i*_*x*(*t* − *τ*_*i*_)*v*(*t* − *τ*_*i*_). To solve this problem, we brought the lemma below which allowed us to write the characteristic equation of (5) in a suitable form that allows the application of classical stability results.

**Lemma 1 .**

For *a*_*i*_ ∈ *R*, we have

(6) $$\begin{array}{c} {\left(\overset{n}{\underset{i=1}{\sum }}a_{i}\right)}^{2}=\overset{n}{\underset{i=1}{\sum }}a_{i}^{2}+2\overset{n-1}{\underset{i=0}{\sum }}\overset{n}{\underset{j=i+1}{\sum }}a_{i}a_{j}. \end{array}$$

**Proof**

This result can be proved easily by induction.

**Remark 1 .**

We note that our approach has the limit to be specific to the model (2); it may not be appropriate for other models. The extension of our method to other models could be considered as one of the perspectives of this work.

### 3.1. Free Equilibrium

System (2) always has a disease-free equilibrium in the form *E*_1_=(*K*, 0,0).

**Proposition 1 .**

If *R*_0_ < 1, then *E*_1_ is locally asymptotically stable.

**Proof**

The Jacobian matrix evaluated at *E*_1_ is

(7) $$\begin{array}{c} J_{E_{1}}=\left(\begin{array}{ccc} -r & -\rho K-r & -\beta K\\ 0 & -\delta & \overset{n}{\underset{i=1}{\sum }}\beta_{i}Ke^{\left(-\lambda \tau_{i}\right)}\\ 0 & b\delta & -\gamma -\beta K \end{array}\right). \end{array}$$

The characteristic polynomial of *J*_*E*_1__ is

(8) $$\begin{array}{c} P_{E_{1}}\left(\lambda \right)=-\left(r+\lambda \right)\left(\lambda^{2}+\lambda \left(\gamma +\beta K+\delta \right)+\delta \left(\beta K+\gamma \right)-\overset{n}{\underset{i=1}{\sum }}\beta_{i}Kb\delta e^{\left(-\lambda \tau_{i}\right)}\right). \end{array}$$

If *τ*_1_=*τ*_2_=⋯=*τ*_*n*_=0, then

(9) $$\begin{array}{c} P_{E_{1}}\left(\lambda \right)=-\left(r+\lambda \right)\left(\lambda^{2}+\lambda \left(\gamma +\beta K+\delta \right)+\delta \left(\beta K+\gamma \right)-\beta Kb\delta \right). \end{array}$$

In this case, the eigenvalues of the matrix *J*_*E*_1__ are

(10) $$\begin{array}{c} \lambda_{1}=-r,\\ \lambda_{2}=\frac{-\left(\gamma +\beta K+\delta \right)-\sqrt{\Delta }}{2},\\ \lambda_{3}=\frac{-\left(\gamma +\beta K+\delta \right)+\sqrt{\Delta }}{2}, \end{array}$$

where Δ=(*γ*+*βK* − *δ*)^2^+4*βKbδ*.

The eigenvalues *λ*_1_ and *λ*_2_ are both negatives for all non-negative parameter values, while the eigenvalue *λ*_3_ can be negative, positive, and zero. For *R*_0_ < 1, we have

(11) $$\begin{array}{c} R_{0}<1\;⟺\;\frac{\beta Kb}{\beta K+\gamma }<1\\ \;⟺\;4\beta Kb\delta <4\delta \left(\beta K+\gamma \right)\\ \;⟺\;4\beta Kb\delta +{\left(\gamma +\beta K\;–\;\delta \right)}^{2}<4\delta \left(\beta K+\gamma \right)+{\left(\gamma +\beta K\;–\;\delta \right)}^{2}\\ \;⟺\;\Delta <{\left(\gamma +\beta K+\delta \right)}^{2}\\ \;⟺\;\lambda_{3}=\frac{–\left(\gamma +\beta K+\delta \right)+\sqrt{\Delta }}{2}<0. \end{array}$$

Hence, all three eigenvalues are negatives. So *E*_1_ is locally asymptotically stable when *τ*_*i*_=0 for (*i*=1,…, *n*).

Now, if *τ*_*i*_(*i*=1,…, *n*) are arbitrary and as *λ*=−*r* is a root of equation (8), we only need to consider

(12) $$\begin{array}{c} \lambda^{2}+\lambda \left(\gamma +\beta K+\delta \right)+\delta \left(\beta K+\gamma \right)-\overset{n}{\underset{i=1}{\sum }}\beta_{i}Kb\delta e^{\left(-\lambda \tau_{i}\right)}=0, \end{array}$$

which is equivalent to

(13) $$\begin{array}{c} \lambda^{2}+\lambda \left(\gamma +\beta K+\delta \right)+\delta \left(\beta K+\gamma \right)=\overset{n}{\underset{i=1}{\sum }}\beta_{i}Kb\delta e^{\left(-\lambda \tau_{i}\right)}. \end{array}$$

If *λ*=*ωi* is a root of equation (12), after substituting and separating real and imaginary parts, we have

(14) $$\begin{array}{c} \left\{\begin{array}{c} -\omega^{2}+\delta \left(\beta K+\gamma \right)=Kb\delta \overset{n}{\underset{i=1}{\sum }}\beta_{i}\cos\left(\omega \tau_{i}\right),\\ -\omega \left(\gamma +\beta K+\delta \right)=Kb\delta \overset{n}{\underset{i=1}{\sum }}\beta_{i}\sin\left(\omega \tau_{i}\right). \end{array}\right. \end{array}$$

Adding the squares of both equations from (14), one has

(15) $$\begin{array}{c} \omega^{4}+\left({\left(\delta +\beta K+\gamma \right)}^{2}-2\delta \left(\beta K+\gamma \right)\right)\omega^{2}+\delta^{2}{\left(\gamma +\beta K\right)}^{2}={\left(Kb\delta \right)}^{2}\left[{\left(\overset{n}{\underset{i=1}{\sum }}\beta_{i}\cos\left(\omega \tau_{i}\right)\right)}^{2}+{\left(\overset{n}{\underset{i=1}{\sum }}\beta_{i}\sin\left(\omega \tau_{i}\right)\right)}^{2}\right]. \end{array}$$

Using Lemma 1 and after algebraic manipulations, equation (15) can also be written in the following form:

(16) $$\begin{array}{c} \omega^{4}+a_{1}\omega^{2}+a_{2}+a_{3}=0, \end{array}$$

where

(17) $$\begin{array}{c} a_{1}={\left(\gamma +\beta K\right)}^{2}+\delta^{2},\\ a_{2}=2{\left(bK\delta \right)}^{2}\overset{n-1}{\underset{i=0}{\sum }}\overset{n}{\underset{j=i+1}{\sum }}\beta_{i}\beta_{j}\left(1-\cos\left(\omega \left(\tau_{i}-\tau_{j}\right)\right)\right),\\ a_{3}=\delta^{2}{\left(\gamma +\beta K\right)}^{2}-{\left(\beta bK\delta \right)}^{2}=\delta^{2}{\left(\gamma +\beta K\right)}^{2}\left(1-R_{0}^{2}\right). \end{array}$$

We have *a*_1_ > 0 and *a*_2_ > 0, and when *R*_0_ < 1, *a*_3_ > 0. Therefore, there is no root *λ*=*ωi*, with *ω* ≥  0 or equation (12), implying that the roots of equation (12) cannot cross the purely imaginary axis. Thus, all roots of equation (12) have a negative part. Then, the equilibrium point *E*_1_ is locally asymptotically stable.

By using a Lyapunov function, we will prove that the equilibrium point *E*_1_ is globally asymptotically stable when *R*_0_ < 1. To study the dynamics of system (2) when *τ*_*i*_ ≥ 0(*i*=1,…, *n*), we need to consider a suitable phase space. For *τ*_*n*_ > 0, we denote by *C*=*C*([−*τ*_*n*_; 0]; *R*^3^) the Banach space of continuous functions mapping the interval [−*τ*_*n*_; 0] into *R*^3^ with the norm ||*φ*(*θ*)||=sup_−*r*_*n*_≤*θ*≤0_ | *φ*(*θ*)| for *φ* ∈ *C*. The non-negative cone of *C* is denoted by *C*^+^=*C*([−*τ*_*n*_, 0], *R*_+_^3^).

**Theorem 1 .**

If *R*_0_ < 1, then *E*_1_ is globally asymptotically stable.

**Proof**

Let *φ*=(*φ*_1_, *φ*_2_, *φ*_3_)=(*x*, *y*, *v*) with *x*(*θ*)=*φ*_1_(*θ*), *y*(*θ*)=*φ*_2_(*θ*), *v*(*θ*)=*φ*_3_(*θ*) for *θ* ∈ [−*τ*_*n*_, 0], consider a Lyapunov function given by

(18) $$\begin{array}{c} V\left(\varphi \right)=b\varphi_{2}\left(0\right)+\varphi_{3}\left(0\right)+b\overset{n}{\underset{i=1}{\sum }}\beta_{i}\int_{-\tau_{i}}^{0}\varphi_{1}\left(s\right)\varphi_{3}\left(s\right)ds. \end{array}$$

The derivative along a solution is given by

(19) $$\begin{array}{c} \dot{V}\left(\varphi \right)=b\left(\overset{n}{\underset{i=1}{\sum }}\beta_{i}\varphi_{1}\left(-\tau_{i}\right)\varphi_{3}\left(-\tau_{i}\right)-\delta \varphi_{2}\left(0\right)\right)+b\delta \varphi_{2}\left(0\right)-\beta \varphi_{1}\left(0\right)\varphi_{3}\left(0\right)-\gamma \varphi_{3}\left(0\right)\\ +b\overset{n}{\underset{i=1}{\sum }}\beta_{i}\left(\varphi_{1}\left(0\right)\varphi_{3}\left(0\right)-\varphi_{1}\left(-\tau_{i}\right)\varphi_{3}\left(-\tau_{i}\right)\right)\\ =b\beta \varphi_{1}\left(0\right)\varphi_{3}\left(0\right)-\beta \varphi_{1}\left(0\right)\varphi_{3}\left(0\right)-\gamma \varphi_{3}\left(0\right)\\ \leq \left(\left(b\beta \;–\;\beta \right)K\;–\;\gamma \right)\varphi_{3}\left(0\right)\\ \leq b\beta K\left(1\;–\;\frac{1}{R_{0}}\right)\varphi_{3}\left(0\right), \end{array}$$

when *R*_0_ < 1, we have $\dot{V}\left(\varphi \right)\;\leq 0$. If $\dot{V}\left(\varphi \right)=0$, then $\left\{\varphi \in R^{3}/\dot{V}\left(\varphi \right)=0\right\}=E_{1}$. The classical LaSalle's invariance Principle implies that *E*_1_ is globally attractive. This confirms the globally asymptotical stability of *E*_1_.

### 3.2. Endemic Equilibrium

Here, we study the stability of the endemic equilibrium point *E*^*∗*^.

**Theorem 2 .**

Equilibrium point *E*^*∗*^ is locally asymptotically stable for *τ*_*i*_ ≥ 0(*i*=1,…, *n*) if the following assumptions are satisfied:

(20) $$\begin{array}{c} \left(A1\right)\;R_{0}>1,\\ \left(A2\right)\;f\left(b\right)<r\gamma +K\rho \gamma \;–\;\beta K\delta ,\\ \left(A3\right)\;K_{i}>0,\;for\;i=1,\ldots ,3, \end{array}$$

where

(21) $$\begin{array}{c} f\left(b\right)=\left(r\gamma +\delta K\left(\beta b-\beta \right)+\rho K\gamma \right)\left(\frac{\gamma K\beta \left(b-1\right)}{K\beta \delta \left(b-1\right)+K\beta \gamma +r\gamma }-\frac{\delta \left(b\;–\;1\right)+\gamma b}{\delta \left(b\;–\;1\right)\left(K\beta b\;–\;\delta \;–\;\beta K\;–\;\gamma \right)}\right),\\ K_{1}=A^{2}-2\left(\tilde{B}+\beta_{0}D\right),\\ K_{2}={\left(\tilde{B}+\beta_{0}D\right)}^{2}-2A\left(C+\beta_{0}E\right)-{\left(\overset{n}{\underset{i=1}{\sum }}\beta_{i}D\right)}^{2}+2\overset{n-1}{\underset{i=1}{\sum }}\overset{n}{\underset{j=i+1}{\sum }}\beta_{i}\beta_{j}D^{2}\left(1-\cos\;\omega \left(\tau_{i}-\tau_{j}\right)\right),\\ K_{3}={\left(C+\beta_{0}E\right)}^{2}-{\left(\overset{n}{\underset{i=1}{\sum }}\beta_{i}E\right)}^{2}+2\overset{n-1}{\underset{i=1}{\sum }}\overset{n}{\underset{j=i+1}{\sum }}\beta_{i}\beta_{j}E^{2}\left(1–\;\cos\;\omega \left(\tau_{i}-\tau_{j}\right)\right), \end{array}$$

and

(22) $$\begin{array}{c} A=\beta x^{*}+\gamma +\delta +\frac{r}{K}x^{*},\\ \tilde{B}=\frac{r}{K}x^{*}\left(\beta x^{*}+\gamma +\delta \right)+\delta \left(\beta x^{*}+\gamma \right)-\delta \beta^{2}x^{*}v^{*},\\ C=\frac{r}{K}x^{*}\delta \left(\beta x^{*}+\gamma \right)-\delta \beta^{2}x^{*}v^{*},\\ D=\left(\frac{r}{K}+\rho \right)x^{*}v^{*}-b\delta x^{*},\\ E=\left(\left(\frac{r}{K}+\rho \right)\gamma +\beta \delta b\right)x^{*}v^{*}-\frac{r}{K}b\delta {\left(x^{*}\right)}^{2}. \end{array}$$

**Proof**

The Jacobian matrix at *E*^*∗*^ is given by

(23) $$\begin{array}{c} J_{E^{*}}=\left(\begin{array}{ccc} -\frac{r}{K}x^{*} & -\left(\rho +\frac{r}{K}\right)x^{*} & -\beta x^{*}\\ \overset{n}{\underset{i=1}{\sum }}\beta_{i}v^{*}e^{\left(-\lambda \tau_{i}\right)} & -\delta & \overset{n}{\underset{i=1}{\sum }}\beta_{i}v^{*}e^{\left(-\lambda \tau_{i}\right)}\\ -\beta v^{*} & b\delta & -\beta x^{*}-\gamma \end{array}\right). \end{array}$$

The characteristic equation associated with *J*_*E*^*∗*^_ is given by

(24) $$\begin{array}{c} \lambda^{3}+A\lambda^{2}+\tilde{B}\lambda +C+\overset{n}{\underset{i=1}{\sum }}\beta_{i}e^{\left(-\lambda \tau_{i}\right)}\left(D\lambda +E\right)=0, \end{array}$$

where $A,\tilde{B},C,D$, and *E* are defined as in Theorem 2.

Considering *τ*_1_=*τ*_2_=⋯=*τ*_*n*_=0, equation (23) becomes

(25) $$\begin{array}{c} J_{E_{1}}=\left(\begin{array}{ccc} -\frac{r}{K}\left(\frac{\gamma }{\beta \left(b\gamma -1\right)}\right) & -\left(\rho +\frac{r}{K}\right)\left(\frac{\gamma }{\beta \left(b\gamma -1\right)}\right) & -\frac{\gamma }{b-1}\\ \beta v^{*} & -\delta & \frac{\gamma }{b-1}\\ -\beta v^{*} & b\delta & -\frac{\gamma b}{b-1} \end{array}\right), \end{array}$$

and equation (24) becomes

(26) $$\begin{array}{c} \lambda^{3}+b_{1}\lambda^{2}+b_{2}\lambda +b_{3}=0, \end{array}$$

with

(27) $$\begin{array}{c} b_{1}=\frac{\left(\delta +\gamma \right)\beta K\left(b-1\right)+\beta \gamma K+r\gamma }{K\beta \left(b\;–\;1\right)},\\ b_{2}=\left[\frac{r\gamma \left(\delta b-\delta +\gamma b\right)2+\gamma b}{\beta K{\left(b\;–\;1\right)}^{2}}+\frac{\delta r\left(K\beta b\;–\;\beta K\;–\;\gamma \right)}{r\gamma \beta +\beta \delta K\left(\beta b\;–\;\beta \right)+pK\gamma }\right]\times \frac{r\gamma +K\rho \gamma -\beta \gamma K}{K\left(b-1\right)},\\ b_{3}=\frac{\delta r\gamma \left(K\beta b\;–\;\beta K\;–\;\gamma \right)}{K\beta \left(b\;–\;1\right)}. \end{array}$$

By the Routh–Hurwitz Criterion, all roots of the polynomial (26) have negative real parts if and only if *H*_1_=*b*_1_ > 0, *H*_2_=*b*_1_*b*_2_ − *b*_3_ > 0, and *H*_3_=*b*_2_*H*_2_ > 0. When *R*_0_ > 1, we have *H*_1_=*b*_1_ > 0 and *b*_2_ > 0. Since *H*_3_=*b*_3_*H*_2_, we only need to consider *H*_2_=*b*_1_*b*_2_ − *b*_3_.

After some algebraic manipulations ([21]) we can prove that

(28) $$\begin{array}{c} H_{2}>0\;⟺\;f\left(b\right)<r\gamma +K\rho \gamma -\beta K\delta . \end{array}$$

So we conclude that when *R*_0_ > 1 and *f*(*b*) < *rγ*+ *Kργ* − *βKδ*, the endemic equilibrium is locally asymptotically stable for *τ*_1_=⋯=*τ*_*n*_=0.

Consider now the case when *τ*_1_,…, *τ*_*n*_ are arbitrary. Finding roots of the equation (24) is impossible explicitly. Instead, we look for the condition under which it has no purely imaginary roots.

Let *λ*=*ωi*(*ω* > 0) be a purely imaginary roots of (24), then

(29) $$\begin{array}{c} -\omega^{3}i-A\omega^{2}+\tilde{B}\omega i+C+\overset{n}{\underset{i=1}{\sum }}\beta_{i}\left(D\omega \;i+E\right)e^{\left(-i\omega \tau_{i}\right)}=0, \end{array}$$

which is equivalent to

(30) $$\begin{array}{c} -\omega^{3}i-A\omega^{2}+\tilde{B}\omega i+C+\overset{n}{\underset{i=1}{\sum }}\beta_{i}\left(D\omega i+E\right)\left(\cos\left(\omega \tau_{i}\right)-i\;\sin\left(\omega \tau_{i}\right)\right)=0. \end{array}$$

Separating real and imaginary parts leads to

(31) $$\begin{array}{c} \left\{\begin{array}{c} A\omega^{2}-C-\beta_{0}E=\overset{n}{\underset{i=1}{\sum }}\beta_{i}\left(E\;\cos\left(\omega \tau_{i}\right)+D\omega \;\sin\left(\omega \tau_{i}\right)\right),\\ \omega^{3}-\tilde{B}\omega -\beta_{0}D\omega =\overset{n}{\underset{i=1}{\sum }}\beta_{i}\left(D\omega \;\cos\left(\omega \tau_{i}\right)-E\;\sin\left(\omega \tau_{i}\right)\right). \end{array}\right. \end{array}$$

Adding the squares of both equations together gives

(32) $$\begin{array}{c} \omega^{6}+\;\left(A^{2}\;–\;2\left(\tilde{B}+\beta_{0}D\right)\right)\omega^{4}+\left({\left(\tilde{B}+\beta_{0}D\right)}^{2}–\;2A\left(C+\beta_{0}E\right)\right)\omega^{2}+{\left(C+\beta_{0}E\right)}^{2}={\left[–\overset{n}{\underset{i=1}{\sum }}\beta_{i}\left(E\;\cos\left(\omega \tau_{i}\right)+D\omega \;\sin\left(\omega \tau_{i}\right)\right)\right]}^{2}={\left[–\overset{n}{\underset{i=1}{\sum }}\beta_{i}\left(E\;\cos\left(\omega \tau_{i}\right)+D\omega \;\sin\left(\omega \tau_{i}\right)\right)\right]}^{2}+{\left[–\overset{n}{\underset{i=1}{\sum }}\beta_{i}\left(D\omega \;\cos\left(\omega \tau_{i}\right)-E\;\sin\left(\omega \tau_{i}\right)\right)\right]}^{2}. \end{array}$$

Using Lemma 1 and after some algebraic manipulations, equation (32) can also be written in the following form:

(33) $$\begin{array}{c} \omega^{6}+K_{1}\omega^{4}+K_{2}\omega^{2}+K_{3}=0, \end{array}$$

where *K*_*i*_(*i*=1,2,3) are as in Theorem 2. If *K*_*i*_ > 0, then all roots of (24) have negative real parts. Hence, the proof is complete.

## 4. Hopf Bifurcation

In this section, we will study the Hopf bifurcation of system (2) but only in the case of one positive term of delay. In fact, it is too difficult to study the general case with *n* > 1, which can be considered as a perspective of this work. Consider *n*=1, then (24) becomes

(34) $$\begin{array}{c} \lambda^{3}+A\lambda^{2}+\tilde{B}\lambda +C+\beta_{0}\left(D\lambda +E\right)=-\beta_{1}\left(D\lambda +E\right)e^{–\lambda \tau_{1}}, \end{array}$$

if *λ*=*ωi* is a root of (34). After substituting and separating real and imaginary parts, we have

(35) $$\begin{array}{c} \left\{\begin{array}{c} -A\omega^{2}+C+\beta_{0}E=-\beta_{1}\left(E\;\cos\left(\omega \tau_{1}\right)+D\omega \;\sin\left(\omega \tau_{1}\right)\right),\\ -\omega^{3}+\tilde{B}\omega +\beta_{0}D\omega =-\beta_{1}\left(D\omega \;\cos\left(\omega \tau_{1}\right)-E\;\sin\left(\omega \tau_{1}\right)\right). \end{array}\right. \end{array}$$

Adding the squares of both equations together gives

(36) $$\begin{array}{c} \omega^{6}+K_{1}\omega^{4}+K_{2}\omega^{2}+K_{3}=0, \end{array}$$

where *K*_1_, *K*_2_, and *K*_3_ are as follows:

(37) $$\begin{array}{c} K_{1}=A^{2}-2\left(\tilde{B}+\beta_{0}D\right),\\ K_{2}={\left(\tilde{B}+\beta_{0}D\right)}^{2}-2A\left(C+\beta_{0}E\right)-{\left(\beta_{1}D\right)}^{2},\\ K_{3}={\left(C+\beta_{0}E\right)}^{2}-{\left(\beta_{1}E\right)}^{2}. \end{array}$$

Denote *ω*_0_ the biggest positive root of (37); then from (35), we have

(38) $$\begin{array}{c} \cos\left(\omega_{0}\tau_{1}\right)=\frac{D\omega_{0}^{4}+\left(AE\;–\;D\left(\tilde{B}+\beta_{0}D\right)\right)\omega_{0}^{2}\;–\;\left(C+\beta_{0}E\right)E}{\beta_{1}\left({\left(D\omega_{0}\right)}^{2}+E^{2}\right)}. \end{array}$$

Let

(39) $$\begin{array}{c} \tau_{1,0}^{j}=\frac{1}{\omega_{0}}\left\{ar\cos\left(\frac{D\omega_{0}^{4}+\left(AE\;–\;D\left(\tilde{B}+\beta_{0}D\right)\right)\omega_{0}^{2}\;–\;\left(C+\beta_{0}E\right)E}{\beta_{1}\left({\left(D\omega_{0}\right)}^{2}+E^{2}\right)}\right)+2j\pi \right\},\;j=1,2,\ldots . \end{array}$$

Then, we can define $\tau^{*}=\underset{j\geq 1}{\min}\tau_{1,0}^{j}$ as the first value of *τ*_1_ when characteristic roots cross the imaginary axis.

Further, differentiating equation (34) with respect to *τ*_1_, we get

(40) $$\begin{array}{c} \left(3\lambda^{2}+2A\lambda +\tilde{B}+\beta_{0}D+\beta_{1}De^{\left(-\lambda \tau_{1}\right)}-\beta_{1}\left(D\lambda +E\right)e^{\left(-\lambda \tau_{1}\right)}\right)\frac{d\lambda }{d\tau_{1}}=\beta_{1}\left(D\lambda +E\right)e^{\left(-\lambda \tau_{1}\right)}. \end{array}$$

This gives

(41) $$\begin{array}{c} {\left(\frac{d\lambda }{d\tau_{1}}\right)}^{-1}=\frac{3\lambda^{2}+2A\lambda +\tilde{B}+\beta_{0}D}{\beta_{1}\left(D\lambda +E\right)e^{\left(-\lambda \tau_{1}\right)}}+\frac{D}{\lambda \left(D\lambda +E\right)}-\frac{\tau_{1}}{\lambda }, \end{array}$$

and after some algebraic manipulations, we get

(42) $$\begin{array}{c} {\left(\frac{d\lambda }{d\tau_{1}}\right)}^{-1}=\frac{2\lambda^{3}+A\lambda^{2}-C-\beta_{0}E}{-\lambda^{2}\left(\lambda^{3}+A\lambda^{2}+\tilde{B}\lambda +C+\beta_{0}\left(D\lambda +E\right)\right)}+\frac{E}{\lambda^{2}\left(D\lambda +E\right)}-\frac{\tau_{1}}{\lambda }. \end{array}$$

Thus,

(43) $$\begin{array}{c} {\left[{\left(\frac{d\lambda }{d\tau_{1}}\right)}^{-1}\right]}_{\lambda =\omega_{0}i}=\frac{-i2\omega_{0}^{3}-A\omega_{0}^{2}-C-\beta_{0}E}{\omega_{0}^{2}\left(-i\omega_{0}^{3}-A\omega_{0}^{2}+\tilde{B}\omega_{0}i+C+\beta_{0}\left(D\omega_{0}i+E\right)\right)}+\frac{-E}{\omega_{0}^{2}\left(D\omega_{0}i+E\right)}-\frac{\tau_{1}}{\omega_{0}i}\\ =\frac{-\left(A\omega_{0}^{2}+C+\beta_{0}E\right)-i2\omega_{0}^{3}}{\omega_{0}^{2}\left(-A\omega_{0}^{2}+C+\beta_{0}E+i\left(-\omega_{0}^{3}+\left(\tilde{B}+\beta D\right)\omega_{0}\right)\right)}+\frac{-E}{\omega_{0}^{2}\left(E+D\omega_{0}i\right)}-\frac{\tau_{1}}{\omega_{0}i}, \end{array}$$

and

(44) Redλdτ1−1λ=ω0i=2ω06−A2−2B˜+β0Dω04−β12E2−C+β0E2ω02−Aω02+C+β0E2+−ω03+B˜+β0Dω02.

So, if *n*=1 and *K*_3_ < 0, there exists a Hopf bifurcation as Re([(*dλ*/*dτ*_1_)^−1^]_*λ*=*ω*_0_*i*_) > 0.

In conclusion, we have the following Hopf bifurcation result.

**Theorem 3 .**

In the case where system (2) has only one no zero delays and if *K*_3_ < 0, then system (2) undergoes a Hopf bifurcation at the endemic equilibrium.

## 5. Numerical Results

In this section, we present numerical simulations to illustrate the various theoretical results previously obtained. Thus, we draw first the curves of system (2) for parameters verifying *R*_0_ less than 1, and we shall do the same for parameters verifying *R*_0_ upper to 1. All simulations are performed using the parameter values in Table 1 are taken from [22].

Since our model considers population of cells, we convert tumor volume to cell population by assuming 1 mm^3^ corresponds to 10^6^ cells [22]. For our numerical simulation, we consider cell populations *x* and y, virus population v is expressed in units of 10^6^, and using the same manner as in [22], we assume that the tumor is completely eliminated, which indicates the total success of the virotherapy, when the total population of tumor cells is reduced to one cell, which means that in the adopted units *u*(*t*)=*x*(*t*)+*y*(*t*)=10^6^.

**Figure 2:**
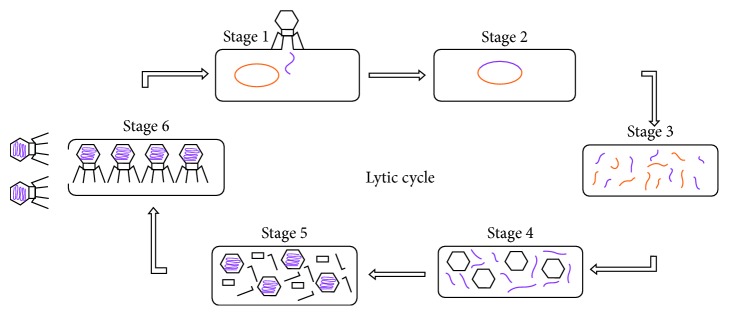
The lytic cycle of oncolytic viruses.

Figure 2 presents the curves of system (2), using various initial conditions when *n*=4 and *R*_0_=0.7. In Section 3, using a suitable Lyapunov function, we have proved that, in this case, *R*_0_ < 1, the disease-free equilibrium *E*_1_ is globally asymptotically stable. From this figure, we see that the curves converge to the free equilibrium *E*_1_, that is the virotherapy fails as the population of tumor cells increase and the population of infected tumor decrease.

**Figure 3:**
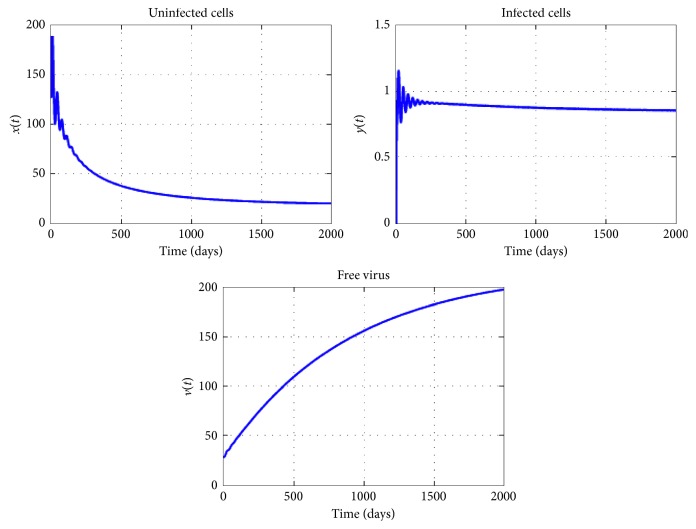
Dynamics of virotherapy when *R*_0_=0.7, *β*_0_=10^−3^, *β*_1_=10^−5^, *β*_2_=2 × 10^−5^, *β*_3_=3 × 10^−5^, *β*_4_=4 × 10^−5^, *τ*_1_=0.2, *τ*_2_=1, *τ*_3_=2, and *τ*_4_=3. The initial conditions are *x*(0)=127, *y*(0)=0, and *v*(0)=30.

Figure 3 provides the curves of system (2) using various initial conditions when *n*=4, *R*_0_=1.5, and the other conditions of Theorem 2 are satisfied. We have theoretically proved in Section 3 by using the technique of stability in a delayed system that the endemic equilibrium *E*^*∗*^ is locally asymptotically stable. From this figure, we see that the curves converge to positive and finite limit, which is the endemic equilibrium. The stability of the equilibrium *E*^*∗*^ implies that a permanent reduction of the tumor load can be reached, even if the virotherapy does not succeed completely.

**Figure 4:**
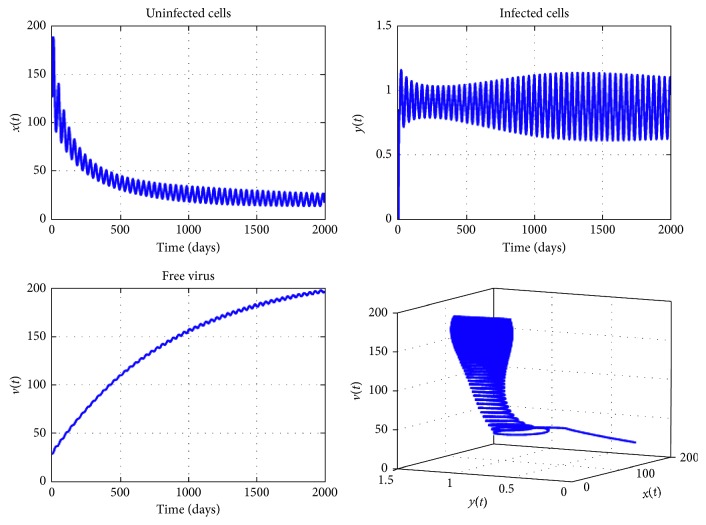
Dynamics of virotherapy when *R*_0_=1,5, *β*_0_=10^−3^, *β*_1_=10^−5^, *β*_2_=2 × 10^−5^, *β*_3_=3 × 10^−5^, *β*_4_=4 × 10^−5^, *τ*_1_=0.2, *τ*_2_=1, *τ*_3_=2, and *τ*_4_=3. The initial conditions are *x*(0)=127, *y*(0)=0, and *v*(0)=30.

**Figure 5:**
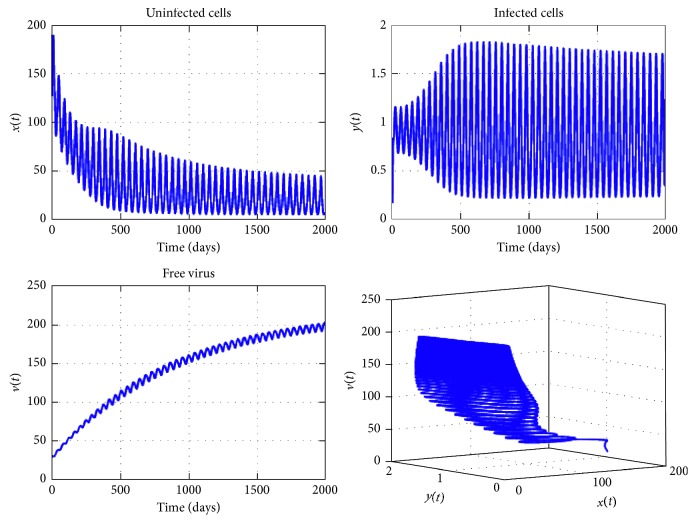
Dynamics of virotherapy when *R*_0_=1,5, *β*_0_=10^−5^, *β*_1_=10^−4^, and *τ*=7 < *τ*^*∗*^=8.8368. The initial conditions are *x*(0)=127, *y*(0)=0, and *v*(0)=30.

**Figure 6:**
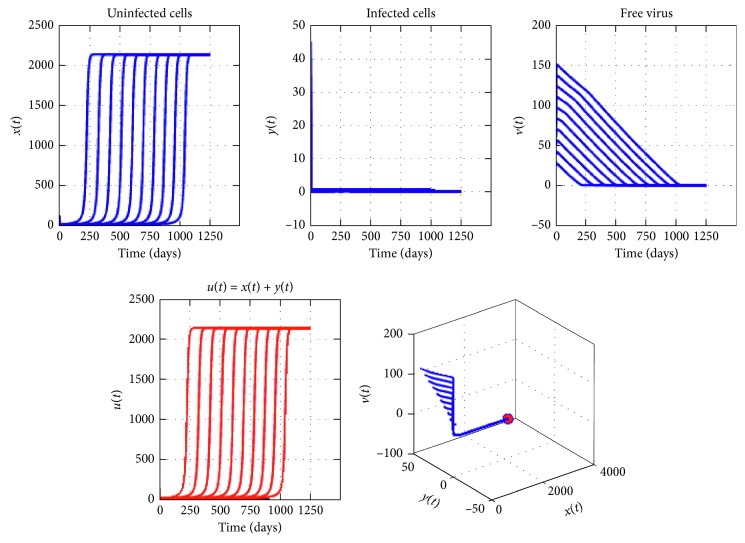
Dynamics of virotherapy when *R*_0_=1,5, *β*_0_=10^−5^, *β*_1_=10^−4^, and *τ*=*τ*^*∗*^=8.8368. The initial conditions are *x*(0)=127, *y*(0)=0, and *v*(0)=30.

**Figure 7:**
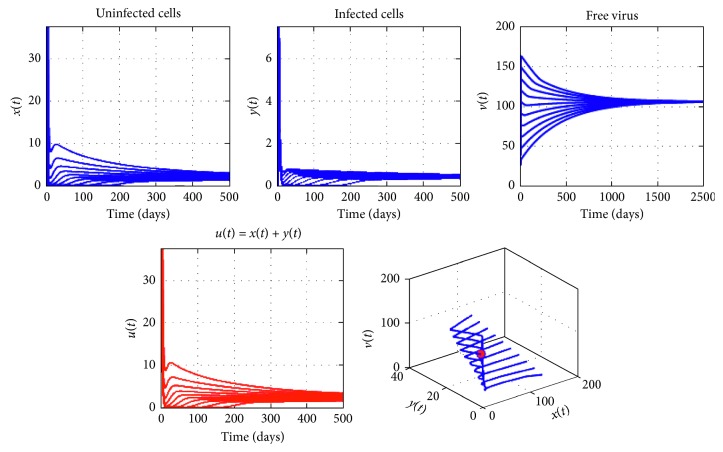
Dynamics of virotherapy when *R*_0_=1,5, *β*_0_=10^−5^, *β*_1_=10^−4^, and *τ*=10 > *τ*^*∗*^=8.8368. The initial conditions are *x*(0)=127, *y*(0)=0, and *v*(0)=30.

Figures 4–6 show that for *τ* < *τ*^*∗*^, the equilibrium point *E*^*∗*^ is asymptotically stable and, for *τ* > *τ*^*∗*^, the equilibrium point *E*^*∗*^ is unstable, and when *τ*=*τ*^*∗*^, a Hopf bifurcation of periodic solutions of system (2) occurs at *E*^*∗*^ (Figure 7).

## 6. Conclusion

The work in this paper contributes to a growing literature on modeling oncolytic virotherapy; we present a mathematical model for the dynamic of oncolytic virotherapy that incorporates multiple time delays representing the multiple time periods to complete the lytic cycle. We give the basic reproductive ratio *R*_0_, and we use it to investigate the stability of the equilibrium states. We prove by formulating suitable Lyapunov function that the disease-free equilibrium is globally asymptotically stable if the basic infection reproduction number *R*_0_ < 1, and when *R*_0_ > 1, the local stability of the endemic equilibrium point depends on function *f*(*b*), representing the replication of the virus in virotherapy and other conditions. Furthermore, we show that there exists a bifurcation value for the lytic cycle period *τ*^*∗*^. For this, if *τ* < *τ*^*∗*^, the positive equilibrium endemic is locally asymptotically stable. The system undergoes a Hopf bifurcation around *τ*=*τ*^*∗*^ and when *τ* > *τ*^*∗*^, the system is unstable. The numerical simulation provides that if *R*_0_ < 1, the virotherapy fails as the population of tumors cells increases and the population of infected tumor decreases, and if *R*_0_ > 1, the virotherapy success and treatment will reach the equilibrium point endemic. The approach that we have introduced with multiple delays is specific to our model or to similar models in other fields. The incorporation of delay from a system that describes virotherapy is an interesting and realistic strategy, and several studies have adopted this method, for example, Wang's work [24].

## Figures and Tables

**Table 1:** Model parameters.

Height parameters	Descriptions	Values
*r*	Growth rate constant	0.206
*K*	Maximal tumor size	2139
*β*_*i*_	Infection rate	Variables
*ρ*	Cell to cell fusion rate constant	0.2145
*δ*	Infected cells death rate	0.5115
*b*	Burst size of a virus	Variables
*γ*	Elimination rate of free virus particles	0.001
*τ*_*i*_	Time to complete lytic cycle variables	Variables

## Data Availability

Te disciplinary data used to support the findings of this study have been deposited in the Network Repository (http://www.networkrepository.com).
